# Oviposition but Not Sex Allocation Is Associated with Transcriptomic Changes in Females of the Parasitoid Wasp *Nasonia vitripennis*

**DOI:** 10.1534/g3.115.021220

**Published:** 2015-10-27

**Authors:** Nicola Cook, Urmi Trivedi, Bart A. Pannebakker, Mark Blaxter, Michael G. Ritchie, Eran Tauber, Tanya Sneddon, David M. Shuker

**Affiliations:** * School of Biology, University of St. Andrews, St. Andrews, KY16 9TH, United Kingdom; † Edinburgh Genomics and Institute of Evolutionary Biology, University of Edinburgh, Edinburgh, EH9 3FL, United Kingdom; ‡ Laboratory of Genetics, University of Wageningen, The Netherlands; § Department of Genetics, University of Leicester, United Kingdom

**Keywords:** sex allocation, transcriptomics, behavior, gene expression, local mate competition

## Abstract

Linking the evolution of the phenotype to the underlying genotype is a key aim of evolutionary genetics and is crucial to our understanding of how natural selection shapes a trait. Here, we consider the genetic basis of sex allocation behavior in the parasitoid wasp *Nasonia vitripennis* using a transcriptomics approach. Females allocate offspring sex in line with the local mate competition (LMC) theory. Female-biased sex ratios are produced when one or a few females lay eggs on a patch. As the number of females contributing offspring to a patch increases, less female-biased sex ratios are favored. We contrasted the transcriptomic responses of females as they oviposit under conditions known to influence sex allocation: foundress number (a social cue) and the state of the host (parasitized or not). We found that when females encounter other females on a patch or assess host quality with their ovipositors, the resulting changes in sex allocation is not associated with significant changes in whole-body gene expression. We also found that the gene expression changes produced by females as they facultatively allocate sex in response to a host cue and a social cue are very closely correlated. We expanded the list of candidate genes associated with oviposition behavior in *Nasonia*, some of which may be involved in fundamental processes underlying the ability to facultatively allocate sex, including sperm storage and utilization.

Linking the evolution of the phenotype to the underlying genotype remains a key aim of evolutionary genetics. To fully understand how natural selection is shaping a given trait, it is necessary to understand the constraints placed on adaptation at the phenotypic level by the underlying genetic architecture (Sokolowski 2001; Orr 2005). In some cases, one or a relatively few genes have provided a good understanding of the genetic basis of a given behavioral trait. One of the best examples is the rover-sitter polymorphism in the foraging strategy of *Drosophila melanogaster* larvae, which is attributable to two alleles of the *foraging* (*for*) gene and is a cGMP-dependent protein kinase (de Belle and Sokolowski 1987; Osborne *et al.* 1997). However, it has been argued that intensive efforts to explain the genetic basis of quantitative traits by identifying underlying genes have yielded rather little (Travisano and Shaw 2013) and that genomic methods such as QTL analysis may leave important small-effect loci undiscovered (Rockman 2011; Travisano and Shaw 2013). For some behavioral traits, the underlying genetic architecture can be very complex with, for example, epistatic effects and pleiotropy to consider (Orr 2005; Radwan and Babik 2012). These complexities of genetic architecture and the polygenic basis of many traits have led to the suggestion that we should focus on evolutionary processes at the phenotypic level instead (Rockman 2011). However, gene-focused and process-focused approaches could be reconciled by studying the genetic basis of quantitative traits where our understanding of phenotypic evolution is already extensive.

Here, we contrast the transcriptomic responses detected at the level of the whole body for two different behaviors related to sex allocation. Our knowledge of how natural selection shapes sex allocation is perhaps more developed than for any other behavioral trait (West 2009). There is a large body of theoretical literature, from the early works of Düsing and Fisher describing how frequency-dependent selection acts on sex allocation (Fisher 1930; Edwards 2000) to the pioneering work of Hamilton (1967) and Trivers and Willard (1973). There have been many refinements to sex allocation theory since (reviewed by West 2009), including the recent work of Macke *et al.* (2011) that explored the ability of individuals to adjust the sex ratio depending on the environment in which they had evolved.

The parasitoid wasp *Nasonia vitripennis* has been the focus of much research on sex allocation behavior. Females of this species allocate the sex of their offspring in line with the local mate competition (LMC) theory (Hamilton 1967; Werren 1980, 1983), which predicts a female-biased sex ratio when mating occurs between the offspring of one or a small number of females on a patch. Briefly, natural selection favors mothers who bias the sex ratio toward their daughters, thereby minimizing competition among her sons for mates and increasing the number of mates available for those sons (Taylor 1981). As the number of females (foundresses) contributing offspring to a patch increases, less female-biased sex ratios are favored. LMC theory has been extended, using *N. vitripennis* as a model, to incorporate a variety of more complex situations, including when the level of LMC on a patch is asymmetrical between broods (Shuker *et al.* 2005), when females are related to their cofoundresses (Shuker *et al.* 2004) and, from a mechanistic point of view, which cues females use to infer the levels of LMC their sons will experience (King *et al.* 1995; Shuker and West 2004). Moreover, females can rapidly alter their pattern of sex allocation when the environment changes (*e.g.*, when other females arrive on a patch) (Shuker *et al.* 2007). In short, we know a lot about sex allocation behavior in *Nasonia vitripennis* at the phenotypic level but little regarding the genetic basis of the behavior, or about how genetic architecture may constrain the evolution of this trait.

Genetic studies of sex ratio in *Nasonia vitripennis* have been largely quantitative. Early work by Orzack and colleagues demonstrated genetic variation for the ability to facultatively adjust sex ratio in line with theoretical predictions (Orzack and Gladstone 1994). More recently, Pannebakker *et al.* (2008) performed a mutation accumulation study, demonstrating that sex ratio has a mutational heritability of 0.001–0.002. The authors calculated that the genetic variation observed in natural populations of *N. vitripennis* is lower than expected given their mutational parameters, suggesting that other selective pressures may be acting against mutations in sex ratio genes, *i.e.*, sex ratio genes may be pleiotropic and have influence over other fitness-related traits. Building upon this hypothesis, Pannebakker *et al.* (2011) identified quantitative trait loci (QTL) associated with sex ratio, which exhibited some overlap with clutch size QTL. These data suggest that perhaps some of the same genes influence both sex ratio and clutch size and have identified genomic locations for further study.

More recently, patterns of differential gene expression between ovipositing and resting *N. vitripennis* females have been uncovered (Pannebakker *et al.* 2013). In particular, oviposition is associated with focusing gene expression away from certain aspects of metabolism during egg-laying. Of the 332 differentially expressed genes identified, the majority were associated with greater expression in resting females. In that experiment, females were alone when ovipositing (*i.e.*, they were single “foundresses”) or resting. Here, we extend this experiment to test whether facultative sex allocation under LMC also leaves a transcriptomic footprint. We explore whole-body gene expression changes under two sets of environmental cues. These cues are used by females to assess the level of LMC experienced by offspring and therefore to allocate sex (Shuker and West 2004). First, we examine gene expression when females oviposit in the presence of cofoundresses (a “social” LMC cue); second, we examine gene expression when females oviposit on either fresh or parasitized hosts, where the presence of eggs is a “host” cue used to facultatively change sex allocation. We therefore determine whether the same genes are upregulated during oviposition when the cues known to influence sex allocation are also available.

## Materials and Methods

### Study species

*Nasonia vitripennis* (Hymenoptera, Chalcicoidea) is a generalist parasitoid of large dipteran pupae including species of Calliphoridae. Depending on host species, females oviposit between 20 and 50 eggs in an individual host, with male offspring emerging just before females (after approximately 14 d at 25°) (Whiting 1967). Male individuals are brachypterous and are unable to fly, remaining close to the emergence site where they compete with each other for emerging females, including their sisters. Females disperse after mating to locate new hosts.

The focal females used in this experiment were from the AsymC strain. This line was originally isolated in 1986 by curing the wild-type strain LabII of *Wolbachia* and is known to be free of sex-ratio distorters (Gherna *et al.* 1991; Werren 1991). Wasps have been maintained on *Calliphora vomitoria* or *C. vicina* hosts at 25°, 16L:8D light conditions ever since. For some experimental treatments cofoundresses were required. These were taken from the red-eye mutant STDR strain, allowing us to track the offspring of a single AsymC female using eye color. The STDR strain is maintained under conditions identical to the AsymC strain.

Importantly, the genome of the AsymC strain has been sequenced and annotated by the Nasonia Genome Working Group (Werren *et al.* 2010), permitting the direct mapping of transcriptomic data from this experiment onto available genomic resources.

### Experimental design

To control for possible host and other maternal effects, we isolated approximately 450 single (2-d-old, mated) wild-type AsymC females from the mass cultures into individual glass vials and provided each with three hosts. We used females from the resulting F1 generation in the experiment, one female per “grandmother.” These experimental females were then provided with a single host for 24 hr as a pretreatment to facilitate egg development. Pretreatment hosts were discarded and each female was then given a piece of filter paper soaked in honey solution for a further 24 hr.

For the transcriptomics experiment, we used a 2 × 3 factorial experimental design, giving a total of six treatment combinations with N = 70 replicates per treatment combination (total N = 420 experimental females). Females were placed into either of two “foundress number” groups: (i) single foundress or (ii) 10 foundress (*i.e.*, one experimental female plus nine red-eye mutant STDR cofoundresses, which allowed us to follow the sex ratios produced by experimental females). Females were then subdivided into one of three “host treatment” groups, in which females were provided with: (i) no hosts; (ii) three fresh hosts; or (iii) three previously parasitized hosts. Group iii hosts had been parasitized for the preceding 24 hr by individual STDR females. Given that females can rapidly alter their sex ratios as the environment changes (Shuker *et al.* 2007), we tested for genes that will alter their expression rapidly upon exposure to these known sex allocation cues (cofoundress number and preparasitized hosts). After a period of 3 hr (the experiment was run between 0900 and 1200, at 25°, 16L:8D light conditions), AsymC females were placed into 1.5-ml microcentrifuge tubes, flash-frozen in liquid nitrogen, and stored at −80° prior to RNA extraction. STDR cofoundresses were discarded and the hosts were retained in the same temperature and light conditions to allow the offspring to develop and eclose normally.

### RNA extraction

Prior to RNA extraction, experimental females exposed to identical treatment combinations were pooled into groups of 10. This resulted in seven pooled samples for each of the six treatment combinations (*i.e.*, in terms of pooled sample, N = 42). Key tissues for the behaviors being tested are not known; therefore, RNA was isolated from whole bodies using the TRIzol Plus RNA purification kit in conjunction with the PureLink RNA mini kit (Life Technologies, Paisley, UK) according to the manufacturer’s instructions. Optional steps for “On-Column PureLink DNase Treatment During RNA Purification” were followed. Concentration and integrity of RNA samples were checked using a Nanodrop Spectrophotometer (Nanodrop Technologies, Wilmington, DE) and a Bioanalyzer system (Agilent Technologies, Santa Clara, CA).

### Library preparation and sequencing

Total RNA samples were prepared for sequencing using the Illumina TruSeq RNA Sample Prep Kit (Illumina, San Diego, CA) following the Illumina TruSeq Sample Preparation v2 (Low Sample) protocol. Briefly, mRNA molecules were purified from total RNA samples using oligo-dT attached magnetic beads and fragmented using divalent cations at 94°. First strand cDNA synthesis was carried out using reverse transcriptase and random hexamer primers. Second strand synthesis was carried out using RNA polymerase I and RNase H. Overhangs resulting from fragmentation were converted to blunt ends and 3′ ends were subsequently adenylated. Sanger indexing adapters were ligated to the fragments that were then purified and PCR-amplified to create the final cDNA libraries for sequencing. Libraries were sequenced on the HiSeq2000 according to the manufacturer’s instructions. Raw sequence reads are available from the Gene Expression Omnibus database at NCBI (Accession: GSE74241).

### Mapping, filtering, and annotation of sequence reads

Reads were aligned to *Nasonia* Official Gene Set version 2 (OGS2, January 2012, http://arthropods.eugenes.org/EvidentialGene/nasonia/genes/) using GSNAP software v2013-02-05. Reads that mapped ambiguously, *i.e.*, to more than one feature, were discarded. Annotation and Gene Ontology information were obtained from the annotation for OGS2 generated by the *Nasonia* Genome Working Group (http://www.hymenopteragenome.org/nasonia/?q=evidential_gene_data). Read counts per sample were obtained using HTseq version 0.5.4p1 using “intersection-strict” mode, whereby the whole read must map to a single transcript to be counted.

In terms of the dataset, we initially obtained 1,296,283,832 reads, of which 95.75% (1,241,156,436) mapped uniquely to OGS2 (see Supporting Information, Table S1 for individual library mapping statistics). Because of the “intersection-strict” mode applied to HTSeq, some of the reads that map ambiguously to a gene were removed and we were left with 155,799,857 reads that mapped to 20,388 genes. Transcripts present at extremely low abundance are a common source of noise in this type of study. Therefore, genes with less than 100 reads across all 42 samples were excluded from further analysis (*i.e.*, a mean of less than three reads per replicate). Removing these very low abundance transcripts left us with 155,652,340 reads from 12,230 genes for the analysis of differential expression (*i.e.*, this last step removed 40.01% of the genes but only 0.09% of the reads).

### Statistical analysis

Tests for differential expression were carried out using DESeq (version 1.12.1) (Anders and Huber 2010) implemented in the R environment (version 3.0.2, R Core Team 2013). As a first processing step, effective library size was estimated using robust regression. These size factors were then used to bring raw read counts per gene to a common scale (normalization). DESeq tests for differential expression within a generalized linear model framework using the negative binomial error distribution to model transcript abundance. Given the 2 × 3 factorial design of this experiment, we made use of the generalized linear model framework to determine which genes were significantly differentially expressed as a result of either of the main effects (foundress number and host type), and also to explore any interaction between the main effects. When there was significant differential expression in association with a main effect, follow-up pairwise tests were performed in DESeq to explore differences between treatment groups. The significance of differential expression is estimated for each gene and then corrected for multiple comparisons using the Benjamini and Hochberg (1995) false discovery rate (FDR) adjustment. Genes were considered to be differentially expressed if, after FDR adjustment, *P* < 0.05.

Correlations in expression changes between host treatment groups were examined using linear regression implemented in the R environment (version 3.0.2, R Core Team 2013).

### Enrichment analysis

Enrichment analysis of Gene Ontology (GO) terms was carried out to determine whether groups of differentially expressed genes are associated with any particular function(s). The analysis was performed using the Blast2GO suite (Conesa *et al.* 2005). The package uses Fisher’s exact test to detect annotation differences between two sets of sequences and corrects for multiple testing using FDR (Benjamini and Hochberg 1995). Groups of differentially expressed genes were compared to *Nasonia* Official Gene Set version 2 to determine whether these groups contained an overrepresentation of any GO terms.

### Data availability

Table S1 contains the mapping statistics for each of the 42 sequenced libraries. Table S2 contains a list of all genes differentially expressed in association with the main effect “host treatment”. Table S3 contains a list of all genes differentially expressed between host treatment A (no hosts) and host treatment B (fresh hosts). Table S4 contains a list of all genes differentially expressed between host treatment A (no hosts) and host treatment C (preparasitised hosts). Table S5 contains a list of all the genes differentially expressed in response to oviposition in both this study and the study carried out by Pannenbakker *et al.* (2013). Table S6 contains a list of genes differentially expressed in association with host treatment that reside within an oviposition QTL significant at the genome-wide level (Pannebakker *et al.* 2011). Table S7 contains a list of genes differentially expressed in response to host treatment that are also differentially expressed in *N. vitripennis* females in response to bacterial infection (Sackton *et al.* 2013). File S1 shows the methods used to carry out additional statistical analysis using only genes know to be differentially expressed in the head of *N. vitripennis* females based on Hoedjes *et al.* 2015). Gene expression data are available at GEO with the accession number: GSE74241.

## Results

Oviposition, *i.e.*, the presence or absence of a host, led to significant changes in gene expression, whereas cues associated with sex allocation did not. In fact, facultative sex allocation cues were not associated with any differential expression in female *N*. *vitripennis* at the whole-body level.

**Figure 1:**
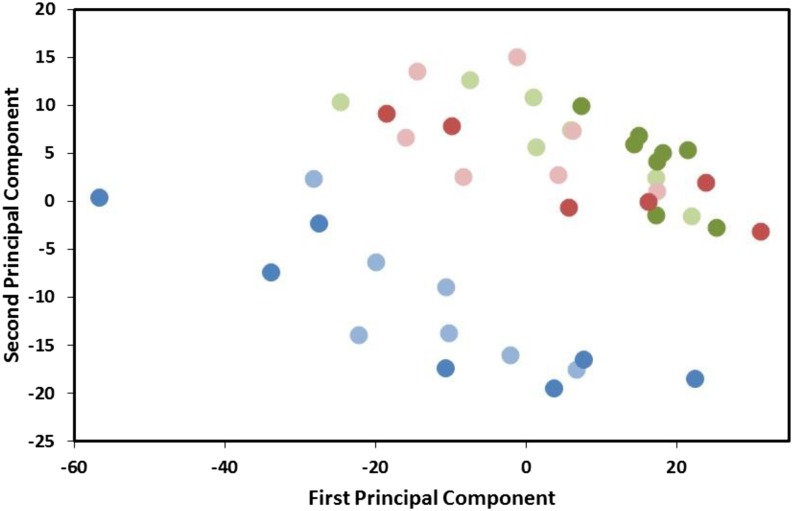
PCA plot. The 42 samples from the RNA-seq study shown in two dimensions by their first two principal components. Blue dots represent samples from the “no host” treatments, green dots represent those from the “fresh host” group, and red dots represent those from the “previously parasitized” treatments. Paler dots represent samples from “single foundress” treatments and darker dots represent samples from “10 foundress” treatments. Samples cluster entirely on the basis of presence (all blue dots) or absence (red and green dots) of a host.

**Figure 2:**
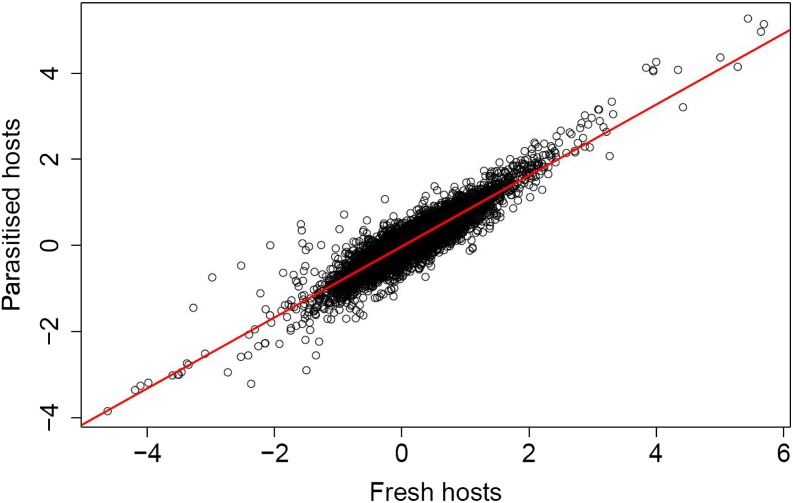
Log_2_ fold change in expression when ovipositing on fresh hosts relative to resting *vs.* that when ovipositing on parasitized hosts relative to resting. Regression line shown in red (R^2^ =0.85, *P* < 0.001).

From a total of 12,230 genes tested, 1359 were differentially expressed in association with the main effect “host treatment” at FDR of *P* < 0.05 (see Figure 1 and Table S2). These differentially expressed genes represent 18,188,035 reads out of a total of 155,652,340, or 11.69% of the total transcriptome. Of these 1359, 28 had total normalized read counts in excess of 100,000 across experimental replicates (Table 1). Importantly, the main effect of host treatment was entirely associated with differences between “no host” and the two other host treatments (Figure 1). In addition, a strong correlation was observed between the expression changes for all 12,230 genes in females ovipositing on fresh hosts (relative to resting females) and females ovipositing on parasitized hosts (relative to resting females) (R^2^ = 0.85, *P* < 0.001) (Figure 2). Pair-wise analyses revealed that in females ovipositing on fresh hosts, 1170 genes (844 of which were upregulated) were differentially expressed relative to the control group (FDR *P* < 0.05) (Table S3). When ovipositing on previously parasitized hosts, 822 genes (540 of which were upregulated) were differentially expressed relative to the control group (FDR *P* < 0.05) (Table S4).

**Table 1:** Genes with more than 100,000 (normalized) reads differentially expressed in association with host treatment in *Nasonia vitripennis* females

**OGS2 ID***^a^*	**OGS2 Description***^b^*	**Normalized Read Count**	***P***	**FDR-Adj. *P***
Nasvi2EG004256	Heat shock protein 83, putative	1330788.29	0.0025	0.0267
Nasvi2EG003712	HEAT shock 70 kD protein cognate 4	439812.48	0	0.0004
Nasvi2EG018266	D-3-phosphoglycerate dehydrogenase	431540.73	0	0
Nasvi2EG013831	Cathepsin L, putative	316827.91	0	0.0001
Nasvi2EG018994	Phosphoenolpyruvate carboxykinase [GTP]	265803.70	0	0
Nasvi2EG000249	Disulfide-isomerase, protein	255728.37	0.0013	0.0159
Nasvi2EG014565	Unknown	247983.80	0.0027	0.0285
Nasvi2EG011426	Trypsin-1	206947.20	0	0
Nasvi2EG014261	Unknown	196083.19	0.0027	0.0285
Nasvi2EG002768	Multifunctional protein ADE2	157350.84	0.0004	0.0062
Nasvi2EG004860	Unknown	152181.24	0.0001	0.0021
Nasvi2EG013799	6-phosphogluconate dehydrogenase decarboxylating	144249.93	0.0026	0.0279
Nasvi2EG010956	dnaJ homolog subfamily A member 1	142950.83	0.0049	0.0455
Nasvi2EG006004	Unknown	133503.67	0	0
Nasvi2EG024534	Heat shock protein cognate 5	132478.99	0	0
Nasvi2EG001276	Cytochrome P450 9e2	128635.36	0	0
Nasvi2EG006840	Nuclear protein 1	128042.56	0	0
Nasvi2EG015454	Unknown	123175.12	0	0.0003
Nasvi2EG003208	Unknown	120639.05	0.0044	0.0419
Nasvi2EG010293	Trypsin-1	120042.75	0.0009	0.0116
Nasvi2EG011806	Proclotting enzyme	115288.61	0.0000	0.0002
Nasvi2EG006008	Ankyrin 2.3/unc44 (Fragment)	114434.65	0	0
Nasvi2EG002346	HEAT shock 70 kD protein cognate 4	113846.10	0	0
Nasvi2EG002769	Amidophosphoribosyltransferase, putative	113668.63	0.0016	0.0193
Nasvi2EG009955	Translocon-associated protein subunit alpha, putative	109362.98	0	0.0001
Nasvi2EG009089	Cyclin-A1	103923.59	0.0035	0.035
Nasvi2EG004595	Myophilin	103854.72	0.0002	0.0035
Nasvi2EG013069	Chemosensory protein	102976.54	0.0053	0.0486

a *Nasonia* Official Gene Set version 2 identifier.

b *Nasonia* Official Gene Set version 2 description.

In terms of sex allocation behavior, there were no differentially expressed genes between those females ovipositing on fresh hosts and those ovipositing on previously parasitized hosts, so that the effects of oviposition itself on gene expression were consistent across host type. There were also no detectable gene expression changes in *Nasonia vitripennis* females in response to the presence of cofoundresses. Thus, neither superparasitism nor the presence of cofoundresses, both of which influence sex allocation, produced gene expression changes detectable at the level of the whole body.

Notably, 11 genes exhibited a greater than four-fold log_2_ change in expression (a common measure of differential expression). Six of these highly expressed genes were upregulated in females ovipositing on either fresh or previously parasitized hosts as compared to “resting” females (Table 2). In addition, one of these genes, Acyl-CoA desaturase, is known to be expressed in the brain (Table 2). However, the enrichment analysis showed that there was no significant enrichment (FDR *P* < 0.1) of any GO terms in the 1359 differentially expressed genes associated with host treatment when compared to the complete *Nasonia* OGS GO annotation. Similarly, there was no significant enrichment of GO terms in those genes differentially expressed between females ovipositing on fresh hosts *vs.* resting females or between females ovipositing on previously parasitized hosts *vs.* resting females.

**Table 2:** Differentially expressed genes with a greater than four-fold log_2_ change in expression compared to control (resting) females

**OGS2 ID***^a^*	**OGS2 Description***^b^*	**Host Treatment Condition**	**Log_2_ Fold Change**	***P***	**FDR-Adj. *P***	**Differentially Expressed in the Brain in Response to Host Conditioning (****Hoedjes *et al.* 2015****) Y/N**
Nasvi2EG016530	Deoxyribonuclease I, putative	Fresh	5.6913	6.11E-26	5.33E-23	N
		Parasitized	5.1398	1.25E-21	8.23E-19	
Nasvi2EG022794	Unknown	Fresh	5.6467	1.14E-31	1.55E-28	N
		Parasitized	4.9640	7.24E-12	1.38E-09	
Nasvi2EG006174	Uricase	Fresh	5.4414	1.65E-55	2.01E-51	N
		Parasitized	5.2696	7.70E-50	3.14E-46	
Nasvi2EG003094	Unknown	Fresh	5.2814	1.07E-09	1.09E-07	N
		Parasitized	4.1488	1.68E-10	2.59E-08	
Nasvi2EG016531	Unknown	Fresh	5.0072	2.06E-27	1.94E-24	N
		Parasitized	4.3667	3.61E-16	1.30E-13	
Nasvi2EG020749	Acyl-CoA desaturase	Fresh	4.3419	1.64E-17	5.15E-15	Y
		Parasitized	4.0783	2.67E-59	3.26E-55	
Nasvi2EG003092	Unknown	Fresh	4.4200	3.08E-11	4.01E-09	N
Nasvi2EG009667	Pacifastin light chain	Parasitized	4.2635	2.22E-40	5.42E-37	N
Nasvi2EG009663	Pacifastin light chain	Parasitized	4.1278	1.78E-49	5.43E-46	N
Nasvi2EG012577	inorganic phosphate cotransporter	Parasitized	4.0746	2.88E-30	2.94E-27	N
Nasvi2EG008011	Unknown	Parasitized	4.0451	2.04E-21	1.25E-18	N

a *Nasonia* Official Gene Set version 2 identifier.

b *Nasonia* Official Gene Set version 2 description.

## Discussion

We have identified 1359 candidate genes associated with oviposition behavior. However, facultative sex allocation by female *Nasonia vitripennis* was not associated with short-term gene expression changes at the whole-body level. This was true for both types of LMC cues tested: *i.e.*, the response to foundress number (a “social” cue) (Shuker and West 2004), the state of the host (parasitized or not: a “host” cue), or a combination of the two.

We considered the possibility that differential gene expression in response to LMC cues may not be detectable using whole-body transcriptomics and that a tissue-specific approach may be required. In particular, gene expression changes in the brain could be of interest as females must detect and process LMC cues to inform sex allocation. To determine whether such tissue-specific signals may have been “drowned out” by our whole-body approach, we made use of a recent study by Hoedjes *et al.* (2015). The authors conditioned *Nasonia* females with exposure to blowfly hosts and contrasted gene expression in the heads of host-conditioned females with that of naïve control females (who were not exposed to hosts) as part of a study on memory formation. We narrowed down our read count data to include only counts from the 1622 genes found to be differentially expressed in *N. vitripennis* heads in response to host-conditioning by Hoedjes *et al.* (2015) and ran our statistical analysis again (File S1). We still found no differential expression in association with the presence of LMC cues (*i.e.*, “foundress number” and preparasitized hosts), but again found 194 genes associated with oviposition (*i.e.*, the presence or absence of a host). We note that of these 194 genes, only 13 were not picked up by our whole-body analysis (*i.e.*, 181 were included in our original analysis). Therefore, it seems that the response to LMC cues is not associated with changes to gene expression. That said, future tissue-specific studies that also examine alternative transcript usage will allow us to interrogate gene expression in yet more detail to clarify this. However, our study has confirmed that oviposition behavior, regardless of the LMC context in which that oviposition takes place, is associated with changes in gene expression. Below we discuss some interesting candidate genes for oviposition and perhaps sex allocation at a more fundamental level.

We have expanded on the set of candidate genes associated with oviposition behavior first generated by Pannebakker *et al.* (2013). Those authors used a tag-based transcriptome sequencing approach (“DeepSAGE”) to identify 232 genes (322 “tags”) that were differentially expressed between ovipositing and resting *N. vitripennis* females. Our RNA-seq approach revealed 1359 candidates, of which 46 overlapped with their list (Table S2 and Table S5). For instance, Pannebakker *et al.* (2013) found *glucose dehydrogenase (gld)* (OGS2 ID: Nasvi2EG010910) to be upregulated in ovipositing females. The GLD protein, expressed in the spermathecal duct, is associated with the storage and utilization of sperm in *Drosophila melanogaster* (Iida and Cavener 2004). We confirmed that *gld* is indeed upregulated in ovipositing females (Table S2, Table S3, Table S4, Table S5) and discovered that a total of eight *glucose dehydrogenase* genes were differentially expressed in ovipositing *vs.* resting females. Given that female *N. vitripennis* need to control sperm release very precisely to allocate sex, we suggest that this gene family may have a role to play in adaptive sex allocation behavior, and we are currently developing functional genomic tests of this hypothesis.

A QTL analysis by Pannebakker *et al.* (2011) detected three sex ratio QTL, one of which was significant at the genome level. Given that sex allocation was not associated with changes in gene expression, it is difficult to make a direct comparison between the results of that study and the results we present here. However, we did find that 104 of the genes differentially expressed during oviposition reside within the QTL significant at the genome-wide level (Table S6). These genes did not vary in expression in response to LMC cues used by females. However, we have confirmed their involvement in oviposition, and further study will reveal whether these genes turn out to be “sex ratio” genes insofar as they are involved in the more fundamental processes underlying the ability to facultatively allocate sex.

Interestingly, we found that the female-specific isoform of the transcription factor *doublesex* (*dsx*) was found to be upregulated in ovipositing females. Differential splicing of male and female *dsx* mRNA generating sex-specific proteins that regulate downstream somatic sexual dimorphism was first described in *Drosophila melanogaster* (Baker and Wolfner 1988). Since then, the *doublesex/mab-3 related* (*Dmrt*) family of transcription factors has been shown to be involved in sex-specific differentiation in all animals studied (Kopp 2012). In *Drosophila*, genes of the sex determination hierarchy, including *dsx* and *fruitless* (*fru*), control the development and differentiation of sex-specific tissues, thereby establishing gender-specific physiology, neural circuitry, and of course behavior (Villela and Hall 2008; Yamamoto and Koganezawa 2013). Dsx also influences sex-specific reproductive behaviors, such as male courtship and female receptivity in *D. melanogaster* (Rideout *et al.* 2007, 2010; Zhou *et al.* 2014), sex-specific morphology, *i.e.*, wing shape in *Nasonia spp*. (Loehlin *et al.* 2010), and mimicry in swallowtail butterflies (*Papilio polytes*) (Kunte *et al.* 2014). Here, we have evidence to support that *doublesex* may be involved in oviposition, another sex-limited behavior.

In addition, we found that three genes annotated as *major royal jelly protein-like* (*mrjpl9*: Nasvi2EG014218, *mrjpl6*: Nasvi2EG021396 and *mrjpl5*: Nasvi2EG014323) and one annotated as *yellow x-2* (Nasvi2EG010358) were differentially expressed in association with “host treatment.” During their first 3 d, honey bee larvae are fed with royal jelly (RJ), a substance secreted by nurse bees and involved in the development of queen larvae (Snodgrass 1925; Buttstedt *et al.* 2014). The nine most abundant proteins in RJ became known as the major royal jelly proteins, MRJPs, now known to have evolved from the *yellow* gene family (Ferguson *et al.* 2011; Buttstedt *et al.* 2014). Evidence suggests that Yellows and MRJPs have diverse and context-dependent roles in development and reproduction (Drapeau *et al.* 2006) and, notably, *Nasonia* has the largest number of *yellow/MRJP* genes so far found in any insect, with a particular expansion of *mrjpls* (Werren *et al.* 2010). Their function in *Nasonia* is still unknown, and Werren *et al.* (2010) note their broad expression across tissues and life stages. Our results here highlight its association with oviposition in *Nasonia vitripennis*.

Enrichment analyses can sometimes provide insight into the possible functions of differentially expressed genes. In the present case, no GO terms were overrepresented, which may be due to the polygenic nature of this trait. However, looking to other studies helped to assign function to some of our candidates. For example, several of our differentially expressed genes are annotated as venom proteins and serine proteases and are likely to be linked to the process of oviposition itself (de Graaf *et al.* 2010) (Tables S2, Table S3, Table S4). Also, we found that 27 genes differentially expressed in *Nasonia vitripennis* individuals responding to bacterial infection (Sackton *et al.* 2013) (Table S7) are also differentially expressed in response to oviposition. These comparisons suggest that some of the genes upregulated as females lay eggs could in fact be “immunity genes” that allow females to guard against bacterial infection posed to them by their dipteran hosts. We note that *glucose dehydrogenase* genes have also been linked with immune response in the silkworm (Sun *et al.* 2012), and further study will confirm whether they serve an immune response and/or a sex allocation function in *Nasonia*. In addition, we found that several of the genes differentially expressed in response to oviposition are annotated as heat shock proteins (HSPs) (Table 1, Table S2, Table S3, Table S4). HSPs, first described in 1962, are highly conserved cellular stress proteins present in all organisms that have known functions in reproduction (Neuer *et al.* 2000). For instance, HSP expression is a crucial process during oogenesis in a number of species, including *Drosophila melanogaster* (Ambrosio and Schedl 1984). Heat shock proteins act as chaperones (mediating folding, assembly, and transport of other proteins) and also in response to cellular stresses, including temperature changes and the presence of viral and bacterial infections (Neuer *et al.* 2000); it is possible that HSPs may be expressed in response to the stresses that a female experiences while interacting with hosts during oviposition.

Previous studies have successfully used transcriptomic approaches to identify candidate genes for sex-specific behaviors. For example, a study by Immonen and Ritchie (2012) identified genes in *Drosophila melanogaster* females that were differentially expressed after only 15 min of exposure to a male courtship song. Gene expression changes were observed in the heads of females in association with stimulation by a courtship song, but a large number of these genes were found to have immune function. Their study confirms that rapid gene expression changes can be induced by short-term exposure to stimuli. However, it also suggests that behavioral transcriptomic studies may pick up immune genes as those most likely to display rapid changes in expression even in a tissue-specific study.

In summary, we did not observe any transcriptomic changes in response to two essential LMC cues, foundress number, and prior parasitism of a host. Future studies targeted to gene expression in specific tissues, for example, the brain, may reveal candidate genes for sex allocation not picked up in this analysis. Using a whole-body RNA-seq approach, we instead revealed some potentially useful mechanisms, highlighted above, that may be involved in sex allocation behavior at a more fundamental level.

## 

## Supplementary Material

Supporting Information
