# Supplementary material for: Oviposition but Not Sex Allocation Is Associated with Transcriptomic Changes in Females of the Parasitoid Wasp Nasonia vitripennis
Source: G3 (Bethesda). 2015 Oct 27;5(12):2885–92. doi: 10.1534/g3.115.021220 (PMC4683659; doi:10.1534/g3.115.021220)
Supplement: Supporting Information [file supp_5_12_2885__index.html]

Oviposition but Not Sex Allocation Is Associated with Transcriptomic Changes in Females of the Parasitoid Wasp Nasonia vitripennis — Supporting Information 

# Oviposition but Not Sex Allocation Is Associated with Transcriptomic Changes in Females of the Parasitoid Wasp *Nasonia vitripennis*

## Supporting Information for Cook *et al.*, 2015

**Files in this Data Supplement:**

- File S1 - Supporting Materials and Methods. (.pdf, 266 KB)
- Table S1 - Mapping statistics for each of the 42 libraries sequenced. (.xlsx, 13 KB)
- Table S2 - Differentially expressed genes associated with the main effect "host treatment". (.xlsx, 105 KB)
- Table S3 - Differentially expressed genes between host treatment A (no hosts) and host treatment B (fresh hosts). (.xlsx, 126 KB)
- Table S4 - Differentially expressed genes between host treatment A (no hosts) and host treatment C (pre-parasitised hosts). (.xlsx, 97 KB)
- Table S5 - Genes differentially expressed in response to oviposition in both the current study and Pannebakker *et al.* 2013. (.xlsx, 12 KB)
- Table S6 - Genes differentially expressed in association with host treatment that reside with an oviposition QTL significant at the genome-wide level (Pannebakker *et al.* 2011). (.xlsx, 14 KB)
- Table S7 - Genes differentially expressed in association with host treatment that are also differentially expressed in *N. vitripennis* females in response to bacterial infection (Sackton *et al.* 2013).
