# Supplementary material for: Oviposition but Not Sex Allocation Is Associated with Transcriptomic Changes in Females of the Parasitoid Wasp Nasonia vitripennis
Source: G3 (Bethesda). 2015 Oct 27;5(12):2885–92. doi: 10.1534/g3.115.021220 (PMC4683659; doi:10.1534/g3.115.021220)
Supplement: Supporting Information [file supp_g3.115.021220_FileS1.pdf]

File S1

Supporting Materials and Methods

Additional differential expression analysis

In their 2015 paper, Hoedjes *et al.* examined differential expression associated with learning in *Nasonia* spp. Differential expression was measured in the heads of both *Nasonia vitripennis* and *Nasonia giraulti* in response to host-conditioning. Host-conditioning is defined in this case as the presentation of a blowfly host and a chocolate odour for 1 hour during which time females were allowed to drill and perform host-feeding. This was followed by a 15 minute resting period and then 15 minutes exposure to a vanilla odour. Gene expression in females conditioned in this manner was then contrasted with a naïve control group.

In Table 1 of their manuscript the Hoedjes *et al.* report 717, 1255 and 1333 transcripts differentially expressed, at 0, 4 and 24 hours respectively, after a host-conditioning treatment. Hoedjes *et al.* provided us with these lists of transcripts and we carried out the following steps:

- Alternative transcript information was discarded.
- Duplicate genes between the three lists were removed.
- 17 transcripts with no OGS2 ID were removed.
- 75 genes with no read count data in the current study were removed.
- This left a total of 1 622 genes know to be differentially expressed in the head of *N. vitripennis* females in response to host-conditioning regardless of timepoint (in the Hoedjes *et al.* study).
- Read counts from the current study for each of these 1 622 genes was compiled.
- Differential expression was examined under a general linear model framework implemented in DEseq as described in the main text for this manuscript.
